# Knee-Cartilage Segmentation and Thickness Measurement from 2D Ultrasound

**DOI:** 10.3390/jimaging5040043

**Published:** 2019-04-02

**Authors:** Prajna Desai, Ilker Hacihaliloglu

**Affiliations:** 1 Department of Biomedical Engineering, Rutgers University, Piscataway, NJ 08854, USA; 2 Robert Wood Johnson Medical School, Rutgers University, New Brunswick, NJ 08873, USA

**Keywords:** wltrasound, knee, osteoarthritis, segmentation, cartilage thickness, local phase

## Abstract

Ultrasound (US) could become a standard of care imaging modality for the quantitative assessment of femoral cartilage thickness for the early diagnosis of knee osteoarthritis. However, low contrast, high levels of speckle noise, and various imaging artefacts hinder the analysis of collected data. Accurate, robust, and fully automatic US image-enhancement and cartilage-segmentation methods are needed in order to improve the widespread deployment of this imaging modality for knee-osteoarthritis diagnosis and monitoring. In this work, we propose a method based on local-phase-based image processing for automatic knee-cartilage image enhancement, segmentation, and thickness measurement. A local-phase feature-guided dynamic-programming approach is used for the fully automatic localization of knee-bone surfaces. The localized bone surfaces are used as seed points for automating the seed-guided segmentation of the cartilage. We evaluated the Random Walker (RW), watershed, and graph-cut-based segmentation methods from 200 scans obtained from ten healthy volunteers. Validation against manual expert segmentation achieved a mean dice similarity coefficient of 0.90, 0.86, and 0.84 for the RW, watershed, and graph-cut segmentation methods, respectively. Automatically segmented cartilage regions achieved 0.18 mm localization accuracy compared to manual expert thickness measurement.

## 1. Introduction

Osteoarthritis (OA) of the knee joint is the most common type of arthritis in elderly people [1]. It occurs when the cartilage between the knee joints starts to degenerate and wears away. Due to this, the bones of the joints glide closely against each other causing pain, lack of mobility between the joints, and swelling. Early detection and improved monitoring is important for the treatment of OA.

Imaging plays an important role during OA detection and management. Currently, X-ray planar radiography is the standard imaging modality used in clinical practice for diagnosing OA and monitoring disease progression [2]. Osteophytes, subchondral cysts, and sclerosis, associated with OA, can be identified from X-ray images. The most common evaluation of radiological OA is the calculation of joint space width (JSW) [2]. The limitation of using X-ray radiography is that it is insensitive to degeneration and lacks the visualization of soft-tissue interfaces such as the cartilage. In order obtain a better understanding of the disease and its progression, various studies, including the Osteoarthritis Initiative (OAI), have exploited Magnetic Resonance Imaging (MRI) for cartilage examination. MRI provides a deeper understanding of early changes in the pathological processes of knee joint. The spin-echo (SE) and gradient-recalled-echo (GRE) imaging sequences are used to obtain morphological information. On the other hand, in order to obtain information about the molecular composition of T2 cartilage mapping, the diffusion-weighted imaging (DWI) and delayed gadolinium enhanced MR imaging of the cartilage (dGEMRIC) sequences were utilized. Traditionally, cartilage thickness, from MRI data, is manually measured by drawing a line between the cartilage region and synovial space. In order to minimize inter- and intra-user variability, segmentation and thickness-measurement computational methods were developed [3,4,5,6,7,8,9,10,11,12]. The routine clinical use of MRI is limited, as it is expensive, has high scanning time, and limited accessibility.

In order to provide a cost-effective and real-time imaging alternative to MRI, ultrasound (US) was investigated to diagnose and monitor cartilage degeneration [13,14,15,16,17,18,19,20]. When compared to MRI, US is inexpensive, can be used to image the joints in multiple planes, is easily accessible, and allows real-time assessment. In US scans, the cartilage region appears to be a monotonous hypoechoic band lying between the soft-tissue interface and bone interface. In Reference [18], a study was carried out to measure cartilage thickness using US, and it compared the results to MRI. Cartilage thickness was assessed from the transverse, anterior, middle, and posterior medial femoral regions. Results showed that US could be used as an alternative clinical tool to measure the relative thickness in posterior and middle medial femoral regions. In another study [19], the authors validated US performance for assessing cartilage thickness using arthroscopic grading as the gold standard. The cartilage was assessed from the medial femoral condyle, sulcus of the femoral condyle, and lateral femoral condyle. This study showed that US scans are a strong indicator of cartilage changes for the early diagnosis of OA. In Reference [20], the authors assessed the deformation of medial femoral cartilage with loaded and unloaded conditions. US scans were acquired in a resting condition (unloaded), and after walking and running (loaded). The study showed that, after loading, there was cartilage deformation, and these subtle changes were captured by US. Manual measurement and qualitative investigation remain the main sources of analysis during OA assessment with US [18,19,20]. However, manual analysis of US data is subject to large inter- and intra-user measurement errors.

As a means of decreasing inter- and intra-user measurement errors, various research groups have focused on developing automated US image-enhancement and cartilage-segmentation methods for accurate and robust cartilage-thickness measurement [21,22,23]. In Reference [21], the authors proposed a new image-processing method, multipurpose beta optimized recursive bihistogram equalization (MBORBHE), for the enhancement of the cartilage region from US images. The proposed framework addresses the limitations of the traditional adaptive histogram method by preserving the information of brightness shift, detail loss, and proper contrast enhancement. Successful cartilage-region enhancement was achieved, but the proposed method also resulted in the enhancement of soft-tissue interfaces that could affect cartilage segmentation and thickness measurement. Recently, a new computational approach, termed as the locally statistical level-set method (LSLSM), was proposed for segmentation or cartilage from 2D knee US data [22]. Segmentation results were validated against other level-set methods, such as the local Gaussian distribution fitting (LGDF) model [24], and locally weighted K-means variational level set (WKVLS) [25]. Quantitative evaluations achieved a mean dice similarity coefficient (DSC) value of 0.91 ± 0.01. Although promising results were achieved, the proposed LSLSM method requires postprocessing of the segmented images using connected component labeling. Successful labeling can only be obtained if segmentation results do not have overlapping regions with the soft tissue and bone interface around the cartilage region. Furthermore, cartilage-thickness measurements were obtained by manual operation using the segmented regions. High levels of noise, low-contrast cartilage scans due to suboptimal alignment of the US transducer with respect to the imaged cartilage, different image-acquisition settings, and anatomical boundaries appearing several millimeters in thickness hamper the success of previously proposed intensity and gradient-based methods.

In order to provide a robust solution to some of these imaging conditions, in this work we propose an intensity-invariant cartilage US image-enhancement and segmentation framework. During the first stage, B-mode US images are enhanced using local-phase-based image features. During the second stage, knee-bone surfaces are automatically localized from enhanced US images using a local-phase image-feature-guided dynamic-programming approach. Localized bone surfaces are used as seeds for automatic segmentation. The final stage involves automatic mean cartilage thickness measurement. We evaluated the performance of three different seed-based segmentation methods. A preliminary study of this approach was reported in Reference [23]. In this paper, we extend our previous work by: (1) validating the proposed framework on a larger dataset, (2) evaluating two additional segmentation methods, and (3) developing an automated cartilage-thickness measurement method.

## 2. Materials and Methods

### 2.1. Data Acquisition

Written consent was obtained prior to the collection of US scans. A total of 200 2D images from 10 healthy volunteers were collected during this study (20 scans per subject). The scans were acquired using a Sonix-Touch US machine (Analogic Corporation, Peabody, MA, USA) with a 14–5 MHz linear US transducer with a depth setting of 3.5 cm and image resolution of 0.15 mm. During the scans, the knee was positioned at 90 deg of flexion, and the US transducer was placed transversely in line with the medial and femoral condyle above the superior edge of the patella. Different scans of the cartilage were obtained from both the left and right knee joints. An ultrasound technician with 20 years of clinical experience collected all the data.

**Figure 1:**
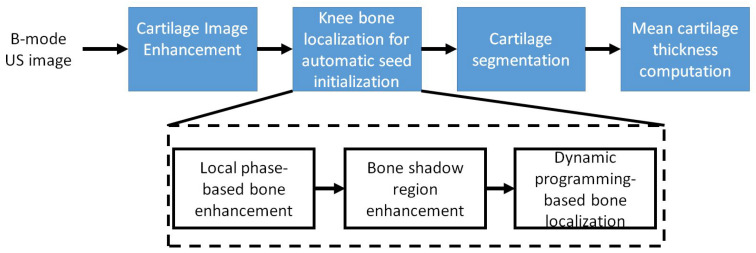
Flowchart of proposed cartilage-segmentation and thickness-measurement method.

The proposed image-processing framework consists of four main subprocesses: (1) cartilage image enhancement, (2) knee-bone localization for automatic seed initialization, (3) cartilage segmentation, and (4) mean thickness computation (Figure 1).

### 2.2. Cartilage Image Enhancement

The orientation of the US transducer with respect to the imaged knee surface and the 3D anatomy of the knee affects the cartilage response profile in the acquired US data. If the transducer is perfectly aligned, and attenuation from the soft-tissue interface is low, then the cartilage interface response profile appears as a dominant ridge edge along the scan-line direction. However, due to the inaccurate alignment of the US transducer, this response profile was degraded during data collection, which affected consecutive image analysis. The first step in our framework involves the enhancement of the low-intensity knee-bone surface and cartilage interface by performing image filtering in a frequency domain similar to [26]:

(1) $$USE\left(x,y\right)=\frac{\sum_{r}\sum_{s}\left[\left[e_{rs}\left(x,y\right)-o_{rs}\left(x,y\right)\right]-T_{r}\right]}{\sum_{r}\sum_{s}\sqrt{e_{rs}^{2}\left(x,y\right)-o_{rs}^{2}\left(x,y\right)}+\epsilon }.$$

Here, e(x,y) and o(x,y) represent the even and odd symmetric filter responses and are obtained by filtering the B-mode US image, US(x,y), using a bandpass quadrature filter in the frequency domain. *r* and *s* represent filter orientation and scale, respectively, and ϵ is a constant used to avoid division by zero. $T_{r}$ is a noise-dependent threshold calculated as a specified number of standard deviations above the mean of local energy distribution because of noise [27]. $T_{r}$ is independently calculated for each orientation.

For the enhancement of bone surfaces, only the absolute response values of odd- and even-filter responses were used to obtain the phase-symmetry metric [27]. However, we were interested in the enhancement of the cartilage response profile, which involves soft tissue and bone boundary. Therefore in our proposed metric, defined in Equation (1), the absolute response values of odd- and even-filter responses are not used. During this work, a 2D Log-Gabor filter is used as the bandpass quadrature filter. The 2D Log-Gabor filter function is defined as [27]:

(2) $$G\left(\omega ,\phi \right)=exp\left[-\frac{{\left(log\left(\omega /\omega_{0}\right)\right)}^{2}}{2{\left(log\left(k/\omega_{0}\right)\right)}^{2}}+\frac{{\left(\phi -\phi_{0}\right)}^{2}}{2\sigma_{\phi }}\right]\;$$

**Figure 2:**
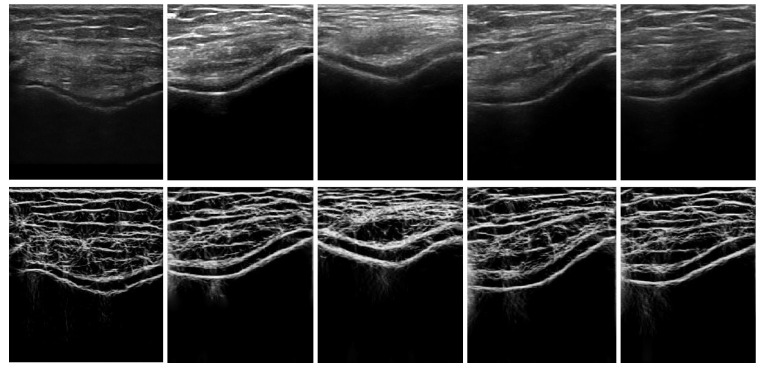
In vivo ultrasound (US) image enhancement: Top row: In vivo B-mode knee-cartilage US image (US(x,y)). Bottom row: Enhanced knee-cartilage US image (USE(x,y)).

In Equation (2), $\sigma_{\phi }=\Delta \phi /s$ evaluates angular bandwidth ΔΩ as, $\Delta \Omega =2\times \sigma_{\phi }\sqrt{2\times log2}$. Δϕ denotes the angular separation between neighboring orientations. Figure 2 shows the enhanced USE(x,y) image, where the bone–cartilage region is enhanced compared to the original B-mode US image. Investigating the results, we can see that the proposed method provides general enhancement results of the cartilage response profile, independent of image intensity. The enhanced image, USE(x,y), is used as an input to the automated knee-bone surface-localization and cartilage-segmentation method, which is explained in the next sections.

### 2.3. Knee-Bone Localization for Automatic Seed Initialization

#### 2.3.1. Local-Phase-Based Bone Enhancement

The enhancement method, explained in the previous section, provides a general enhancement method for soft-tissue, cartilage-region, and bone-surface response, where the intensity values for all these regions are represented with high intensity values (Figure 2). Therefore, using enhanced image USE(x,y) as an input to the dynamic programming approach results in the localization of features that do not correspond to bone surface, resulting in wrong segmentation for the cartilage region. To gain enhancement with minimum soft-tissue and cartilage interface, and more bone representation, three image phase features (local-phase tensor (LPT(x,y)), local weighted mean phase angle (LwPA(x,y)), and local-phase energy (LPE(x,y))), were calculated. LPT(x,y) is a tensor-based local-phase feature-extraction method providing general enhancement, independent of the specific bone edge response profile. LPT(x,y) is obtained using [26]:

(3) $$LPT\left(x,y\right)=\sqrt{T_{even}^{2}+T_{odd}^{2}}\times cos\left(\phi \right).$$

In Equation (3) ϕ represents the instantaneous phase indicating the local contrast independently of feature type, and $T_{even}$ and $T_{odd}$ represent the symmetric and asymmetric feature responses that are defined as [28]:

(4) $$\begin{array}{c} T_{even}=\left[H(US_{DB}\left(x,y\right))\right]{\left[H(US_{DB}\left(x,y\right))\right]}^{T},\\ T_{odd}=-0.5\times (\left[\nabla US_{DB}\left(x,y\right)\right]{\left[\nabla \nabla^{2}US_{DB}\left(x,y\right)\right]}^{T}+\\ \left[\nabla \nabla^{2}US_{DB}\left(x,y\right)\right]{\left[\nabla US_{DB}\left(x,y\right)\right]}^{T}). \end{array}$$

Here *H*, ∇, and $\nabla^{2}$ denote the Hessian, gradient, and Laplacian operations. $US_{DB}\left(x,y\right)$ is obtained by masking the band-pass filtered USE(x,y) image with a distance map. The masking operation results in the enhancement of bone surfaces located deeper in the image, as opposed to soft-tissue artefacts closer to the transducer surface.

The LPE(x,y) and LwPA(x,y) image features are computed using monogenic signal theory. The monogenic signal image, denoted as $US_{M}\left(x,y\right)$, [26,29] is formed by combining α-scale space derivative quadrature band-pass (ASSD) filtered LPT(x,y) image and Riesz filtered component as:

(5) $$US_{M}\left(x,y\right)=\left[LPT_{B}\left(x,y\right),LPT_{B}\left(x,y\right)\times h_{1}\left(x,y\right),LPT_{B}\left(x,y\right)\times h_{2}\left(x,y\right)\right].$$

In Equation (5), $h_{1}\left(x,y\right)$ and $h_{2}\left(x,y\right)$ represents the spatial domain vector valued Riesz filter. $LPT_{B}\left(x,y\right)$, is bandpass filtered LPT(x,y) image. ASSD filters are used as bandpass filters, as they have shown improved edge detection in US images [29,30]. LPE(x,y) and LwPA(x,y) are defined as:

(6) $$LPE\left(x,y\right)=\sum_{sc}\left|US_{M1}\left(x,y\right)\right|-\sqrt{US_{M2}^{2}\left(x,y\right)+US_{M2}^{3}\left(x,y\right)};$$

(7) $$LwPA\left(x,y\right)=\arctan\frac{\sum_{sc}US_{M1}\left(x,y\right)}{\sqrt{\sum_{sc}US_{M1}^{2}+\sum_{sc}US_{M2}^{2}\left(x,y\right)}}.$$

In Equation (7), sc represents the number of scales. LPE(x,y) denotes the underlying shape of the bone boundary, and LwPA(x,y) preserves all the structural details of US image. The final local-phase bone image (LP(x,y)) is obtained by combining all the three phase features as

(8) LP(x,y)=LPT(x,y)×LPE(x,y)×LwPA(x,y).

**Figure 3:**
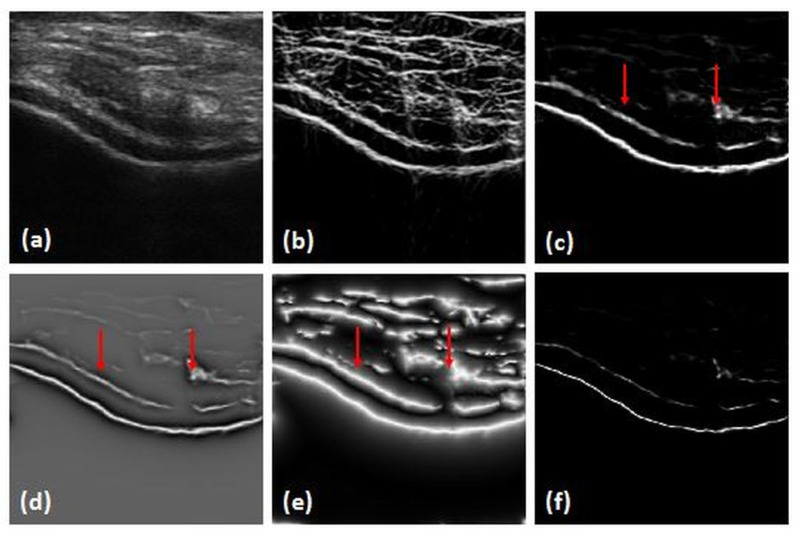
Local-phase image bone features: (**a**) original B-mode US(x,y). (**b**) Enhanced US image USE(x,y). (**c**) Local-phase tensor image (LPT(x,y)). (**d**) Local-phase energy image (LPE(x,y)). (**e**) Local weighted mean phase angle image (LwPA(x,y)). (**f**) Local-phase bone image (LP(x,y)). Red arrows point to extracted soft-tissue interfaces where enhancement was achieved.

The combination of the three phase feature images results in the suppression of soft-tissue interfaces while keeping bone surfaces more compact and localized (Figure 3). LP(x,y) is used for the extraction of bone-shadow regions from the US data.

#### 2.3.2. Bone-Shadow Enhancement

Acoustic bone-shadow information in US is important during bone imaging. Real-time feedback of bone-shadow information can guide the clinician to a standardized diagnostic viewing plane with minimal artefacts, and can provide additional information for bone localization. The proposed bone-shadow region enhancement method is based on the confidence-map (CM) approach [31] using an LP(x,y) image. The framework is modeled using US signal scattering and attenuation information that are combined as [30]:

(9) $$CM_{LP}\left(x,y\right)=US_{A}\left(x,y\right)BSE\left(x,y\right)+\left(1-US_{A}\left(x,y\right)\right)\rho$$

In Equation (9), $CM_{LP}\left(x,y\right)$ is the CM image of local-phase bone image LP(x,y) obtained using Reference [31]. $US_{A}\left(x,y\right)$ is the US signal-transmission map, ρ is an echogenicity constant of the tissue surrounding the bone. BSE(x,y) denotes the enhanced bone-shadow image. The $US_{A}\left(x,y\right)$ is minimized using the below function:

(10) $$\frac{\lambda }{2}\|US_{A}\left(x,y\right)-CM_{LP}{\left(x,y\right)\|}_{2}^{2}+\sum_{j\in x}\|W_{j}o\left(D_{j}*US_{A}\left(x,y\right)\right){\|}_{1}$$

Here, *o* represents elementwise multiplication, *x* is an index set, and * is convolution operator. $W_{j}$ is a weighting matrix calculated as $W_{j}\left(x,y\right)=exp(-|D_{j}\left(x,y\right)*CM_{LP}\left(x,y\right){|}^{2})$. $D_{j}$ is computed using higher-order differential filters that enhance bone features in local regions while suppressing image noise. BSE(x,y) is computed using $US_{A}\left(x,y\right)$ as:

(11) $$BSE\left(x,y\right)=[\left(CM_{LP}\left(x,y\right)-\rho \right)/{\left[max\left(US_{A}\left(x,y\right),\epsilon \right)\right]}^{\delta }]+\rho$$

**Figure 4:**
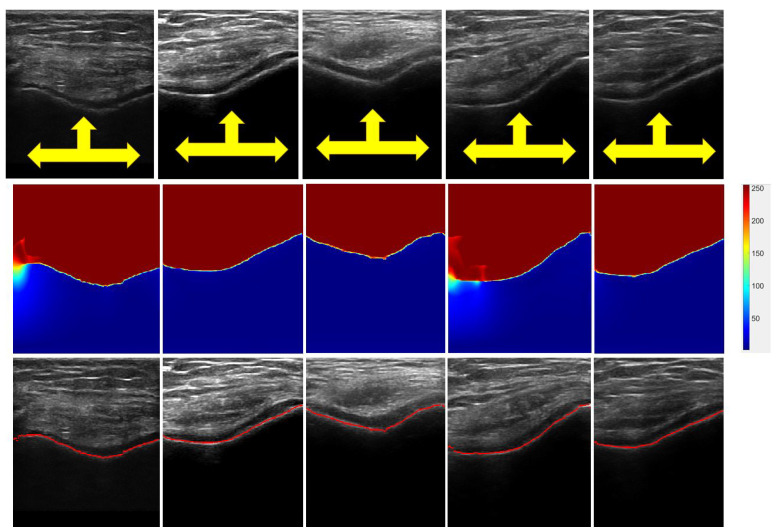
Bone-surface localization results. Top row: B-mode in vivo US knee scans. Yellow arrows show bone-shadow regions. Middle row: Enhanced bone-shadow image BSE(x,y) obtained by processing B-mode US scans shown in top row. Soft-tissue interface, red color coding. Bone-shadow regions, blue. Intensity values depict the probability of a signal reaching the transducer imaging array if the signal propagation started at that specific pixel location. The transition region between the soft-tissue and bone-shadow regions represent the expected bone-shadow interface. Bottom row: Localized bone surfaces, shown in red, overlaid on the B-mode US scans.

In Equation (11), δ is the tissue attenuation coefficient, and ϵ is a constant used to avoid division by zero. Figure 4 displays various obtained BSE(x,y) images from corresponding B-mode US images. Investigating BSE(x,y) images, we can see a clear separation between the soft-tissue interface and shadow region with minimal intensity variations in both regions. Intensity values depict the probability of a signal reaching the transducer imaging array if signal propagation started at that specific pixel location. Furthermore, BSE(x,y) shows a clear transition from the soft-tissue interface to the bone surface by depicting sharp intensity change between two interfaces (Figure 4). The BSE(x,y) and LP(x,y) images are used during bone-surface localization, which is explained in the next section.

#### 2.3.3. Bone-Surface Localization Using Dynamic Programming

**Figure 5:**
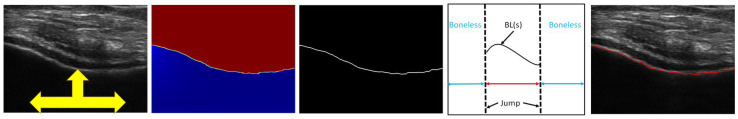
Bone-surface localization. (**a**) In vivo B-mode US knee scan. Yellow arrow, bone-shadow region. Enhanced bone-shadow image BSE(x,y). Soft-tissue interface, red color. Bone-shadow regions, blue. Intensity values depict the probability of a signal reaching the transducer imaging array if the signal propagation started at that specific pixel location. The transition region between the soft-tissue and bone-shadow regions represent the expected bone-shadow interface. (**b**) Bone probability image. (**c**) Bone, boneless, and jump regions. (**d**) Localized bone surface, shown in red, overlaid on original B-mode US image.

Localization of the bone surface within a column *s*, denoted as *BL(s)*, is achieved by minimizing a cost function composed of two energy functions, internal energy ($E_{int}\left(x,y\right)$) and external energy ($E_{ext}\left(x,y\right)$). $E_{int}\left(x,y\right)$ is determined by masking LP(x,y) image with BSE(x,y), which provides a probability map of where the expected bone surface is located (Figure 5b). $E_{ext}\left(x,y\right)$ is obtained by dividing the US image into three regions marked as the bone region, boneless region, and jump region, i.e., the region between the two; these regions are defined as:

(12) $$E_{ext}\left(i,j\right)=\left\{\begin{array}{cc} \nu \|\frac{dBL}{ds}{\|}^{2}+\xi \|\frac{d^{2}BL}{ds^{2}}{\|}^{2}+\varsigma & Bone\;region\\ JumpCost & Jump\;region\\ \nu D_{1}^{2}+\xi D_{2}^{2} & Boneless\;region \end{array}\right.$$

In the above equation, ν and ξ are the weights of smoothness and curvature. ς is a negative scalar to ensure bone connectivity. BL(s) is minimized using local-phase-based image guided dynamic programming as:

(13) $$BLmin\left(i,j\right)=E_{int}\left(i,j\right)+min_{k}\left[BLmin\left(k,j-1\right)+E_{ext}\left(k,j\right)\right],$$

Here, BLmin(i,j) denotes the minimum cost function moving from first column to the pixel in the $i^{th}$ row and $j^{th}$ column, and *k* represents the row index of the image. During optimization, the index of pixel *k,j* with its minima is stored in the following function: $Indexmin\left(i,j\right)=argmin_{k}\left[BLmin\left(k,j-1\right)+E_{ext}\left(k,j\right)\right]$. Localization of the bone surface is obtained by tracing back from the last column of the US image using:

(14) $$BL_{opt}\left(s\right)=\left\{\begin{array}{cc} NR+1 & s=NC\\ Indexmin[s+1,BL_{opt}\left(s+1\right)] & s=1,\ldots ,(NC-1) \end{array}\right.$$

In Equation (14), $BL_{opt}$ is the optimized localization path where the energy-cost function is minimized. The number of columns and rows of the B-mode US image are denoted as *NC*, and *NR*. The last column and row in the US image are also indicated using *NC* and *NR*. The mean bone-surface localization accuracy of this method was reported to be 0.26 mm [26]. Qualitative results of the localized knee-bone surfaces are displayed in Figure 4 and Figure 5. In the next section, we explain how these localized bone surfaces are used as seed points for automated cartilage segmentation.

### 2.4. Cartilage Segmentation

In this paper, we investigate three different seed-based segmentation methods, random walker (RW), watershed, and graph-cut, as they showed better performance with prior shape knowledge. RW segmentation is advantageous over the nonsmoothness of the boundaries (metrication error), preference for shorter boundaries (shrinking bias), boundary length regularization, and number of initial seeds [32,33,34,35]. Watershed is widely used in medical-image segmentation because of its ease of use, lower computing time, and complete division of images with low contrast and weak boundaries. The segmented results provide closed contours, thus eliminating postprocessing such as contour joining [32,36,37,38,39]. The graph-cuts have also extensively been employed for medical-image segmentation due to their accuracy and robustness [40,41]. Below, we first show how localized bone surfaces, explained in the previous section, are used as initial seed points to segmentation algorithms. Following this, we provide a brief explanation on each investigated segmentation method. During the segmentation process, enhanced US data USE(x,y) are segmented. In order to investigate the improvements achieved by using USE(x,y) images as an input to segmentation, we also performed segmentation using the original B-mode US data.

#### 2.4.1. Seed Initialization

The ideal seed points for the above mentioned segmentation methods must lie inside the region and should be near the center of the region of interest. The distance from the foreground seed pixel to its neighboring pixels should be small enough to allow continuous growing. Automatically extracted bone surfaces are used as initial seeds for automatic cartilage-segmentation algorithms. In Reference [42] mean cartilage knee thickness, obtained from 11 cadavers using a surface probe, had a range from 1.69 to 2.55 mm (Mean: 2.16 ± 0.44 mm). Therefore, mean knee-cartilage thickness value, denoted as MKT, was used to automatically initialize the seeds for the validated segmentation algorithms.

For the RW segmentation algorithm, background regions were initialized by translating localized bone surfaces 2×MKT toward the bone-shadow region and the soft-tissue region above the cartilage. Foreground regions were initialized by translating localized bone surface MKT÷2 toward the cartilage region in the direction of the US transducer. For the watershed algorithm, internal markers were initialized with the translation of MKT÷2, and the external marker was initialized on the localized bone surface and with the translation of 2×MKT above the cartilage region. For the graph-cut algorithm, foreground seeds were marked by translating the localized bone surfaces by MKT÷2 and background seeds, with the translation of 2×MKT above and below the cartilage region. The obtained cartilage segmentations using the initialized seed values were qualitatively validated in ten US scans obtained from one of the volunteer subjects (Subject 1), and were were kept constant throughout quantitative validation.

#### 2.4.2. Random-Walker Image Segmentation

In RW, the input image is represented as graph G=(V,E), where *V* corresponds to pixels and *E* are the edges connecting each pair of adjacent pixels [33]. Edges are weighted based on the pixel intensities and gradient values such that the edge with the highest gradient value is weighted more. Weighted function $w_{ij}$ is given as:

(15) $$w_{ij}=exp\left(-\beta {\left(g_{i}-g_{j}\right)}^{2}\right);\forall_{\left(i,j\right)}=1\cdots N;i\neq j.$$

Here, $g_{i}$ and $g_{j}$ are the pixel intensities at each pixel $v_{i}$ and $v_{j}$, and β is a constant parameter used to normalize square gradients ${\left(g_{i}-g_{j}\right)}^{2}$. The user labels pixels as foreground and background, and each unlabeled pixel releases a random walk, which is classified based on the probability values of each unlabeled pixel reaching the labeled pixel. The probability for each unlabeled pixel $x_{U}$ is calculated as:

(16) $$\left(L_{U}+\gamma I_{U}\right)x_{U}=-B^{T}x_{S}+\gamma \lambda .$$

where *L* represents the Laplacian of the graph, *I* is the identity matrix, *x* is the probability vector of each pixel, λ is an optional vector of prior probabilities weighted by γ, and U,S denotes unlabeled and labeled seeds.

#### 2.4.3. Watershed Image Segmentation

In the watershed algorithm, the gray image is transformed as a topographic relief. The objective of watershed transform is to find the `catchment basins’ and `watershed ridge lines’ that divide the neighboring catchment basin in the image [38]. In a traditional watershed algorithm, a hole is punched in each of the local minima of the relief, and the entire topography is flooded from below the relief by letting the water through the hole rising at a uniform rate. When the rising water in the catchment basin is about to merge, a dam is built around the basin to stop the merging. These dam boundaries corresponds to the dividing lines of watershed.

A marker-controlled watershed algorithm is an enhancement of the traditional watershed algorithm which defines a marker and a segmentation function for efficient segmentation of objects with boundaries expressed as ridges. Markers are placed as an internal marker (foreground) associated with the region of interest, and external marker (background) associated with the backgrounds. In traditional watershed, the catchment basin of image function *f* is defined as $X_{h_{max}}$ obtained after the recursion of the following function:

(17) $$\begin{array}{c} X_{h_{min}}=T_{h_{min}}\left(f\right)\\ X_{h+1}=MIN_{h+1}\cup IZ_{T_{h+1}\left(f\right)}\left(X_{h}\right),\;\;h_{min}\leq h<h_{max} \end{array}$$

In the above equation, $X_{h_{min}}$ is the set of points of image I, $T_{h}$ is the threshold, $MIN_{h+1}$ is the union of all regional minima at h+1, I is a 2D grayscale image with values in interval $\left[h_{min},h_{max}\right]$. In a marker-based watershed, we impose minima to image function *f* at specific locations denoted as Markers (*M*). New image function *g* is defined as

(18) $$g\left(p\right)=\left\{\begin{array}{cc} h_{min-1} & if\;p\in M\\ f(p) & otherwise \end{array}\right.$$

Here, *p* represents the pixel co-ordinates, and $h_{min-1}$ represents a new value dedicated to initial markers. The new recursion function is given as

(19) $$\begin{array}{c} X_{h_{min-1}}=T_{h_{min-1}}\left(g\right)\\ X_{h+1}=IZ_{T_{h+1}\left(g\right)}\left(X_{h}\right),\;\;h_{min-1}\leq h<h_{max} \end{array}$$

#### 2.4.4. Graph-Cut Image Segmentation

The graph-cut segmentation algorithm [40] is similar to the RW, where the input 2D image is represented as an undirected graph G=(V,E), defined as the set of nodes and of undirected edges (*E*), where each pair of the connected node is represented by a single edge e=(p,q)∈E. The graph consists of two special terminal nodes *S*(source), and *T*(sink) that represents the foreground and background labels. Each edge e∈E is assigned non-negative weight $w_{e}$. The cut divides the nodes between the terminals where s−t is a subset of edges C∈E, such that terminals *S* and *T* are separated as G=(V,E/C). The cost of cut is given as the sum of weights on edges, which is represented as

(20) $$\left|C\right|=\sum_{e\in C}w_{e}$$

### 2.5. Automatic Cartilage-Thickness Computation

**Figure 6:**
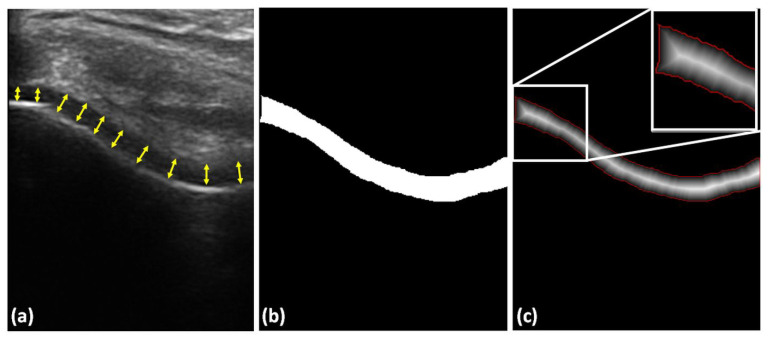
Cartilage-thickness measurement. (**a**) Example manual thickness measurement using 10 anatomical landmarks obtained by drawing a normal line between cartilage–bone interface and the synovial space, shown with yellow arrows. (**b**) Automatically segmented cartilage. (**c**) Distance map obtained from the segmented image shown in (**b**). Red pixels, cartilage boundary, used during the calculation of mean cartilage thickness. White rectangle, zoomed-in region for improved display.

In order to automatically measure cartilage thickness, we calculate the Euclidean distance map from the segmented cartilage region. The distance values corresponding to the automatically extracted cartilage boundary were averaged for the final thickness calculation. This analysis was repeated for manually segmented and all automatically segmented cartilage regions during quantitative validation. We also performed a second manual operation by drawing a normal line between the cartilage–bone interface and the synovial space on original B-mode US images at ten different points and the mean thickness was computed for each B-mode US image (Figure 6). Figure 6 shows an example distance-map image, and the extracted cartilage boundary used during thickness calculation.

Automatically segmented cartilage regions and thickness values were compared with manual segmentation and thickness measurements provided by an expert ultrasound technician. Segmentation validation was obtained by calculating DSC. Automatically computed thickness values were compared with manually measured expert thickness values. We also provide quantitative and qualitative results if B-mode US data were used as input to segmentation methods rather than the enhanced USE(x,y) image. The proposed method was implemented in MATLAB R2017a software package, and ran on a 3.40 GHz Intel® CoreTM i7-4770 CPU, 16 GB RAM Windows PC.

Parameter settings: The Log-Gabor filter was designed using the filter parameters provided in Reference [27]. LPT(x,y) images were calculated using the filter parameter values defined in Reference [28]. Bone-shadow enhancement was achieved using λ = 2. Tissue echogenicity constant ρ was chosen as 90% of the maximum intensity value of $CM_{LP}\left(x,y\right)$ image. η = 2, β = 90, and γ = 0.03 were set as constant to obtain CM(x,y) and $CM_{LP}\left(x,y\right)$ images. For bone localization, ν = 50, ξ = 100, ς = 0.15, Jumpcost = 0.8, $D_{1}=D_{2}=$ 1 were set as constant values [26]. The parameters for bone-surface localization and bone-shadow enhancement were previously validated on 150 US scans collected from 7 subjects. Therefore, we did not change these parameters and adapted the same values reported in Reference [26]. During qualitative and quantitative analysis, all parameter values mentioned in this section were kept constant.

## 3. Results

### 3.1. Cartilage-Segmentation Qualitative Results

**Figure 7:**
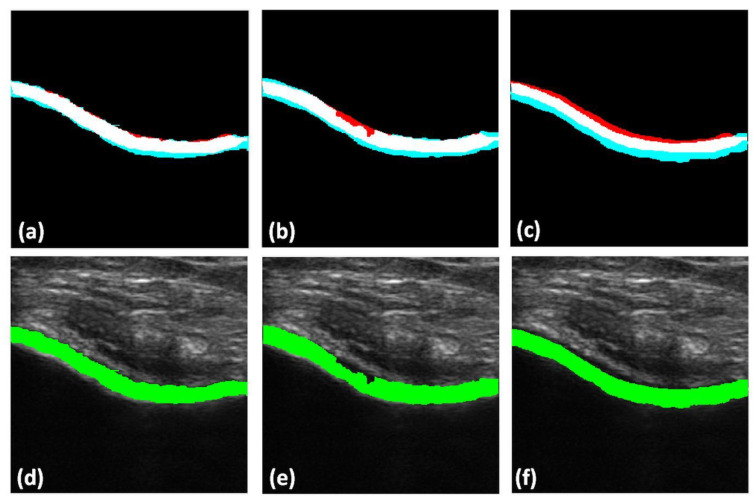
Top row: Qualitative results of automatically segmented cartilage when using USE(x,y) as input to the segmentation method, overlaid on the expert manual segmentation (red: false negative, magenta: false positive, white: true positive): (**a**) Manual segmentation overlaid with random-walker (RW) segmentation. (**b**) Manual segmentation overlaid on watershed segmentation. (**c**) Manual segmentation overlaid on graph-cut segmentation. Bottom row: Automatically segmented cartilage region overlaid on original B-mode US data: (**d**) Cartilage region segmented using RWmethod. (**e**) Cartilage region segmented using watershed method. (**f**) Cartilage region segmented using graph-cut method.

**Figure 8:**
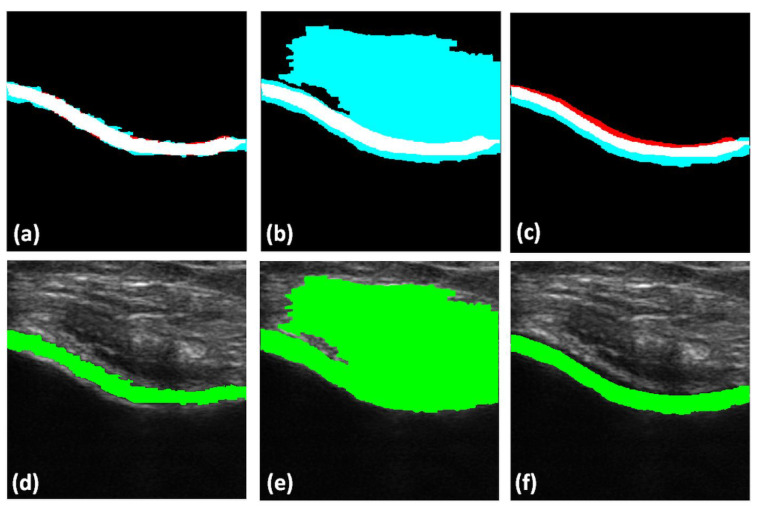
Top row: Qualitative results of automatically segmented cartilage using B-mode US data as an input to the segmentation method, overlaid on expert manual segmentation (red: false negative, magenta: false positive, white: true positive): (**a**) Manual segmentation overlaid with RW segmentation. (**b**) Manual segmentation overlaid on watershed segmentation. (**c**) Manual segmentation overlaid on graph-cut segmentation. Bottom row: automatically segmented cartilage region overlaid on original B-mode US data: (**d**) Cartilage region segmented using RW method. (**e**) Cartilage region segmented using watershed method. (**f**) Cartilage region segmented using graph-cut method.

Qualitative results of the automatically segmented cartilage regions using the three different automatic segmentation methods and the manual expert segmentations are shown in Figure 7. Investigating the results, we can infer that the RW algorithm yielded better cartilage segmentation, whereas the watershed and graph-cut algorithms are limited by over- and undersegmentation for various cartilage sections. Figure 8 shows the qualitative results of cartilage segmentation obtained when the original B-mode images were used as an input to the segmentation methods. Qualitative results show that the RW algorithm yielded better cartilage segmentation, whereas watershed and graph-cut were limited by oversegmentation. Comparing the qualitative results, shown in Figure 7 and Figure 8, we can see the improvements achieved in segmentation quality when using the enhanced US images USE(x,y) as an input to the investigated segmentation methods.

### 3.2. Cartilage-Segmentation Quantitative Results

Average computational time for segmentation using RW, watershed, and graph-cut was 11.08 (±0.2), and 10.53 and 11.51 (±0.3) seconds, respectively. These computation times include the required time for the image-enhancement and bone-surface localization steps.

Table 1 shows the mean DSC for all three different segmentation algorithms investigated during this work. Overall, the RW method obtained a higher mean DSC value compared to the watershed and graph-cut segmentation algorithms. The mean DSC was 0.90, 0.86, and 0.84 for the RW, watershed, and graph-cut methods, respectively (Table 1).

In Table 1, we also report the average recall, precision rates, and F-scores for the three different segmentation methods. RW achieved the the best performance compared to the other two methods. When using original B-mode US data as an input to the segmentation methods, the DSC decreased to 0.79, 0.65, and 0.76 for the RW, watershed, and graph-cut methods, respectively (Table 1). The lower F-score, precision, and recall values further suggest that the algorithm returned less relevant results as compared to the enhanced US images (USE(x,y)).

### 3.3. Cartilage-Thickness Measurement Quantitative Results

Table 2 shows the mean and standard deviation for computed cartilage thickness. The results indicate that the RW segmentation algorithm is more reproducible to manual-segmentation results as compared to the watershed and graph-cut methods. Quantitative results also indicate that there is a 0.15 mm difference between the obtained thickness measurements using manual landmark selection and manual segmentation. This difference also shows that there is a variation in manual measurements. This is an expected result due to manual labeling of US data being an errorprone procedure.

**Figure 9:**
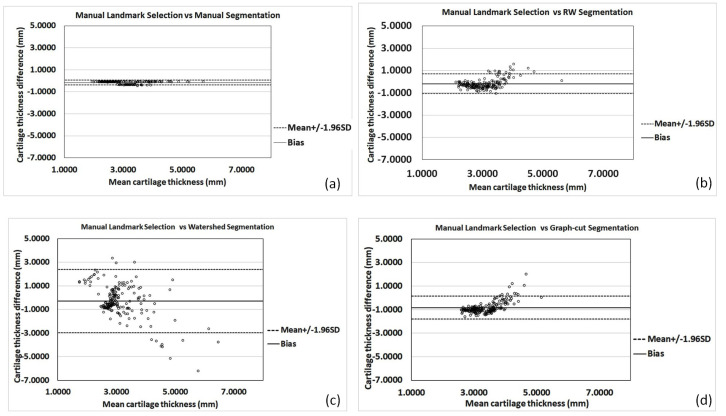
Bland–Altman plots for thickness comparison obtained with the (**a**) manual thickness computation, (**b**) RW, (**c**) watershed, and (**d**) graph-cut methods.

The Bland–Altman plots shown in Figure 9 display a comparison of cartilage thickness obtained by manual anatomical landmark selection from B-mode US data and the thickness values computed using the investigated methods, as well as the measured thickness from manually segmented cartilage regions. The mean error, difference between the manual landmark-based thickness calculation,, and all investigated thickness computations, were −0.15 mm (±0.11 mm), −0.18 mm (±0.45 mm), −0.28 mm (±1.36 mm), and −0.83 mm (±0.49 mm) for the manual segmentation, RW, watershed, and graph-cut methods, respectively. Investigating Table 2 and Figure 9, we could identify that the automatic RW-based cartilage-thickness method achieved the closest thickness-measurement results from the investigated automatic methods to the manual landmark-based thickness measurement.

A paired t-test between manual landmark-based cartilage-thickness measurements and measurement obtained from manual segmentation, RW, watershed, and graph-cut segmentation methods at a 5% significance level achieved *p* values as shown in Table 3 (first row). Investigating the results, we can see that the measurements have significant differences. A reason for this can be attributed to the difference between the number of used landmarks, 10 during this work, and the number or pixels corresponding to the boundary of the segmented cartilage. In order to investigate this, we performed a second significance analysis. The same t-test was performed between thickness measurement obtained from the manual segmentation and measurements obtained from RW, watershed, and graph-cut segmentation methods. The achieved *p* values are shown in Table 3 (second row). Results show that the RW and watershed thickness values have no significant difference. Statistical-significance results between the three automatic-segmentation methods using a paired t-test with 5% significance achieved p values < 0.05, showing that there is significant difference.

## 4. Discussion and Conclusions

Knee-cartilage region segmentation and thickness analysis from 2D US scans has potential for the clinical assessment of cartilage degeneration, a clinical indication used for OA diagnosis and monitoring. We presented a fully automatic and accurate method for cartilage image enhancement, segmentation, and thickness measurement from 2D US data. Quantitative evaluations demonstrated that there was no significant agreement between manual landmark-based cartilage-thickness measurement, and thickness measured from manually segmented cartilage regions. During this work, we evaluated three different segmentation methods. The overall qualitative and quantitative results indicate that, between the RW, watershed, and graph-cut algorithms, RW segmentation is more consistent with the manual results. Quantitative evaluations showed that there is no significant agreement between manual landmark-based cartilage thickness measurement, and thickness measured from manually segmented cartilage regions. This further proves the manual segmentation process of US data is an errorprone procedure. Furthermore, manual measurements and segmentations were performed by a single experienced US technician. Intra- and inter-user variability errors need to be evaluated in order to fully understand the challenges involved during the manual segmentation process. In order to fully overcome the errors introduced during the manual segmentation of US data, gold-standard thickness measurements obtained from an MRI scan should be investigated. Furthermore, thickness calculations were performed using the distance function. A more accurate thickness computation method is the star-line-based method proposed in Reference [43], which we aim to investigate as part of our future work.

The proposed framework requires bone-surface translation to mark the initial seeds for the segmentation algorithm. The seeds were translated on the basis of prior shape knowledge of healthy cartilage and were kept constant for the whole dataset during validation. The method will still be successful for segmenting cartilage from subjects with slight-to-moderate OA with thinned (but connected) cartilage. For segmenting broken cartilages associated with severe OA, automatic seed initialization might be problematic. However, since seed extraction is based on the localization of knee-bone surfaces, the seed-selection process is not affected by the severity of the OA. More in-depth analysis is necessary in order to assess the full clinical usability of the proposed work for segmenting cartilage regions from OA patients.

The quality of cartilage segmentation depends on the collected image data and seed initialization for the segmentation algorithm. As US is user-dependent modality, an important consideration while evaluating articular cartilage is the inclination and the positioning of the US transducer on the proper plane. During data collection, specific attention was given to collect clinically adequate knee scans. In the future, we plan to develop methods based on deep learning for automatic adequate scan plane selection. In order to improve accuracy and robustness, we plan to extend our work for processing 3D US scans. Recently, medical-image segmentation methods based on deep-learning theory have had successful results. Further comparison of deep-learning-based segmentation methods is required in order to assess the full potential of the proposed framework.

In this work, we were interested in the development of a general cartilage enhancement and segmentation method that could be applied to any B-mode US image collected from a standard US machine or point-of-care US device for widespread applicability in a standard clinical setting. In recent years, researchers have been looking into designing segmentation or enhancement methods based on extracted information from raw radio-frequency (RF) US data. Although access to RF data is only available in dedicated research machines, it appears that RF signal information could provide important information about the cartilage and should be further investigated. Elastography and shear-wave elastography (SWE) has also been investigated for imaging cartilage [44,45]. In Reference [44], the authors mention that strain mapping cartilage regions using a static compression method is challenging, and optimization of the technique is required. For SWE, generation and measurement of mechanical waves in cartilage tissue is problematic [46]. Commercially available US machines with SWE imaging capabilities are optimized to detect Young’s modulus values less than 0.3 MPa, which is less than the required limit for imaging cartilage [46]. Therefore, the new wave of propagation models should be investigated in order for SWE to be successfully employed for cartilage imaging.

## Figures and Tables

**Table 1:** Quantitative validation of segmentation results. Dice similarity coefficient (DSC), precision, and recall rates for the investigated segmentation methods when using enhanced (USE(x,y)) and B-mode US (US(x,y)) data as input to the segmentation methods.

Quantitative results when using enhanced US image USE(x,y).				
Method	DSC Mean ± SD	Precision	Recall	F-score
RW	0.90 ± 0.01	0.88	0.92	0.86
Watershed	0.86 ± 0.04	0.82	0.91	0.86
Graph-cut	0.84 ± 0.03	0.81	0.87	0.84
Quantitative results when using B-mode US image US(x,y).				
Method	DSC Mean ± SD	Precision	Recall	F-score
RW	0.79 ± 0.1	0.80	0.80	0.79
Watershed	0.65 ± 0.2	0.60	0.78	0.66
Graph-cut	0.76 ± 0.09	0.72	0.82	0.76

**Table 2:** Quantitative results for automatic cartilage-thickness measurement.

Method	Image	Mean ± SD (mm)
Manual measurement	Original B-mode	2.95 ± 0.66
Automatic measurement	Manual Segmentation	3.1 ± 0.68
	RW Segmentation	3.14 ± 0.46
	Watershed Segmentation	3.23 ± 1.21
	Graph-cut Segmentation	3.78 ± 0.35

**Table 3:** Statistical significance results between manual and automated cartilage-thickness measurements.

	Manual Segmentation	RW	Watershed	Graph Cut
Manual landmark-based segmentation	0.02	0.001	0.004	0.000003
Manual Segmentation	Not Applicable	0.57	0.2	0.00002
